# S17. ZNF804A GENE AND CANNABIS USE: INTERACTION ON THE RISK FOR PSYCHOSIS IN A NON-CLINICAL SAMPLE

**DOI:** 10.1093/schbul/sby018.804

**Published:** 2018-04-01

**Authors:** Jordi Soler Garcia, Barbara Arias, Jorge Moya, Manuel Ignacio Ibañez, Generós Ortet, Lourdes Fañanás, Mar Fatjó-Vilas

**Affiliations:** 1 University of Barcelona, Biomedicine Institute of the University of Barcelona (IBUB); 2 University of Barcelona, Biomedicine Institute of the University of Barcelona (IBUB), Centre for Biomedical Research Network on Mental Health (CIBERSAM); 3 University of Lleida, Centre for Biomedical Research Network on Mental Health (CIBERSAM); 4 Universitat Jaume I, Centre for Biomedical Research Network on Mental Health (CIBERSAM)

## Abstract

**Background:**

The ZNF804A gene and cannabis use are risk factors for psychosis, both of which have also been associated with schizotypic traits. This study aimed to test whether ZNF804A (rs1344706) modulates the relation between cannabis use and schizotypy levels in a general population sample.

**Methods:**

The sample consisted of 389 Spanish non-clinical subjects (43% males, mean age=21.1(2.19)). Schizotypy was evaluated with the three factors of the Schizotypal Personality Questionnaire-Brief (SPQ-B): Cognitive-Perceptual (CP), Interpersonal (I) and Disorganized (D). Subjects were classified as cannabis users or non-users. Multiple linear regressions were conducted to test the effect of genetic and environment factors and their interaction on SPQ-B scores. Sex and anxiety scores (evaluated with SCL) were included as covariates.

**Results:**

The analyses showed a significant linear relationship between the ZNF804A and SPQ-I: homozygotes AA showed higher scores (p=0.001). An interaction between cannabis use and rs1344706 on SPQ-CP was observed: among individuals AA, cannabis users presented higher scores than non-users, while among individuals CC, cannabis users presented lower scores compared to non-users (p=0.005).

**Discussion:**

These results add evidence on that the ZNF804A modulates schizotypy and suggest that schizotypy levels are influenced by an interaction between the exposure to cannabis and the ZNF804A genotype.

